# Histopathological assessment of laterality defects in zebrafish development

**DOI:** 10.1080/19768354.2021.1931443

**Published:** 2021-05-26

**Authors:** Md. Ashraf Uddin Chowdhury, Ahmed A. Raslan, Eunhye Lee, Juneyong Eum, Byung Joon Hwang, Seung-Hae Kwon, Yun Kee

**Affiliations:** a Department of Biomedical Science, College of Biomedical Science, Kangwon National University, Chuncheon, Republic of Korea; b Department of Pharmacy, International Islamic University Chittagong, Chattogram, Bangladesh; c Department of Molecular Bioscience, College of Biomedical Science, Kangwon National University, Chuncheon, Republic of Korea; d Seoul Center, Korea Basic Science Institute, Seoul, Republic of Korea; e Division of Biomedical Convergence, College of Biomedical Science, Kangwon National University, Chuncheon, Republic of Korea

**Keywords:** Zebrafish, laterality defect, heterotaxy, cardiovascular histopathology, parapineal

## Abstract

Laterality defects during embryonic development underlie the aetiology of various clinical symptoms of neuropathological and cardiovascular disorders; however, experimental approaches to understand the underlying mechanisms are limited due to the complex organ systems of vertebrate models. Zebrafish have the ability to survive even when the heart stops beating for a while during early embryonic development and those adults with cardiac abnormalities. Therefore, we induced laterality defects and investigated the occurrence of situs solitus, situs inversus, and situs ambiguus in zebrafish development. Histopathological analysis revealed heterotaxy in both embryos and juvenile fish. Additionally, randomization of left-right asymmetry of the brain and heart in individual zebrafish embryos under artificial experimental pressure further demonstrated the advantage of transparent zebrafish embryos as an experimental tool to select or reduce the embryos with laterality defects during early embryonic development for long-term studies, including behavioural and cognitive neuroscience investigations.

## Introduction

Left-right asymmetric organs, such as the brain and heart, require differential information to set the asymmetric position of each organ in early embryonic development. Genetic and non-genetic factors, as well as environmental disruption, cause laterality defects recapitulating various left-right asymmetry manifestations, including dextrocardia, d-transposition of the great arteries, left-sided superior vena cava, malrotation of the gut, and transposition of the outflow tract, reflecting congenital cardiovascular disorders. Heterotaxy or situs ambiguus is a congenital left-right asymmetry defect that affects the arrangement of major visceral organs in the chest and abdomen (Sutherland and Ware 2009; Anderson et al. 2018). Asymmetric information comes from various sources, such as asymmetries in the embryo-scale genetic cascades, including the left-sided Nodal cascade, organ-intrinsic mechanical forces, and cell-level chirality; however, the relative influence of these sources and how they collaborate to drive asymmetric morphogenesis remain unclear (Grimes et al. 2020).

Zebrafish have a two-chambered heart. The heart forms a tube and is positioned left of the midline by 23 hours post-fertilization (hpf) and undergoes dextral or rightward looping by 36 hpf to produce a heart, with the ventricle to the right of the atrium, hereby correctly positioning the heart chambers relative to one another during embryonic development (Desgrange et al. 2018). Three heart tube morphologies occur in zebrafish: dextral loop (D-loop), leftward loop (L-loop), or unloop. Zebrafish is limited to exploring the phenotypes specific to vertebrates with four chambers; however, its advantage of better survival from cardiac defects through embryonic development allows the exploration of detailed gene function during development and the histopathology of visceral organs over the lifetime (Stainier et al. 1996; Ki et al. 2019).

Cognitive and behavioural left-right asymmetries are common in vertebrates, and defects in the development of brain asymmetry have been implicated in various diseases (Kong et al. 2020). Zebrafish is a valuable model organism that allows for a greater understanding of the genetic and developmental processes that give rise to brain asymmetry (Concha et al. 2012). The epithalamus shows evolutionarily conserved asymmetries within both the pineal complex and adjacent habenular nuclei (Concha and Wilson 2001). The pineal complex of zebrafish consists of a pineal organ that is located in the midline and a left-sided parapineal organ at 2 dpf at the same dorsal level as the pineal organ during larval development (Concha et al. 2000; Liang et al. 2000; Gamse et al. 2003).

Laterality defects such as cardiac looping appear during zebrafish development, and it is possible to detect abnormal morphology during embryonic development to a certain extent using conventional microscopy as well as advanced imaging technologies including single plane illumination microscopy as previously applied in the lab (Park et al. 2015; Eum et al. 2016; Ki et al. 2019). Here, we examined the combinatorial laterality defects of the parapineal and cardiac looping in early embryos by inducing the laterality defect under experimental pressure and investigated the relationship between the brain and heart in the establishment of left-right asymmetry. We also demonstrated the occurrence of situs solitus, situs inversus, and situs ambiguus of visceral organs during embryonic development, as well as in 5-week-old juvenile fish.

## Materials and methods

### Zebraﬁsh lines and maintenance

Wild type AB andAB*zebraﬁsh (*Danio rerio*) and transgenic linesTg*(foxd3:EGFP)* (Gilmour et al. 2002), *Tg(myl7:EGFP)* (Moro et al. 2012), and *Tg(fli1:EGFP)* (Lawson and Weinstein 2002) were used in this study. Adult zebraﬁsh were raised in balanced salt water at 27.5°C in a 14/10 h light/dark cycle. Embryos were obtained by natural spawning of adult zebrafish, raised in E3 medium (5mM NaCl, 0.33 MgSO_4_, 0.33mM CaCl_2_, and 0.17 KCl) in an incubator at 28.5°C, and staged by hours post-fertilization (hpf) and days post-fertilization (dpf) (Kimmel et al. 1995). The experimental protocols used in this study were evaluated and approved by the Animal Use and Ethics Committee of the Kangwon National Universityand performed according to the Institutional Animal Care and Use Committee guidelines.

### Induction of laterality defects

The embryos were obtained from the fish that went through four or five consecutive generations of sibling mating or treated at 22.5°C from the 16-cell stage (1.5 hpf) to the 16-somite stage (17 hpf), transferred back to a 28.5°C incubator, and raised until examined. Laterality defects of the pineal complex and heart were investigated in embryos and 5-week-old juvenile fish.

### Wholemount in situ hybridization

Whole mount *in situ* hybridization was performed as previously described (Thisse and Thisse 2008; Choe et al. 2020) with some modifications. Anti-sense RNA probes were prepared using a digoxigenin– or fluorescein-labeling mix (Roche, Basel, Switzerland). To generate *dand5* and *lft2* RNA probes, 700–800 base pairs of each DNA fragment were amplified from zebrafish cDNA and cloned into pBluescript KS+ vector using oligonucleotide primers (Park et al. 2019). The *foxa3* and the *myl7* probe constructs were kindly provided by Prof. Donghun Shin from the University of Pittsburgh School of Medicine, USA and Prof. Hyunju Ro from the Chungnam National University, Republic of Korea, respectively.

### Stereomicroscope imaging of zebrafish embryos

The embryos were anaesthetized with 0.02% tricine (Sigma-Aldrich, St. Louis, MO, USA) in E3 medium and mounted with 3% methyl cellulose (Sigma-Aldrich) in a confocal dish (SPL Life Sciences, Pocheon, Republic of Korea). Fluorescence imaging of the transgenic embryos was performed using a SZX16 stereoscope (Olympus, Tokyo, Japan) with an AxioCam GRC camera (Carl Zeiss, Oberkochen, Germany).

### Single plane illumination microscopy

The zebrafish *Tg(foxd3:EGFP)* transgenic line was used for imaging the pineal complex in the brain, *Tg(myl7:EGFP)* for imaging the myocardial cells in embryonic myocardium, and *Tg(fli1:EGFP)* for imaging the endothelial cells of the embryonic endocardium as described previously (Park et al. 2015; Eum et al. 2016). Each embryo was first anaesthetized in E3 media containing 0.02% tricaine, embedded in 1.5% low-melting point agarose, and visualized in the imaging chamber at 27°C throughout the imaging period. Three-dimensional (3D) single plane illumination microscopy (SPIM) imaging was performed using a Lightsheet Z.1 fluorescence microscope with a W Plan-Apochromat 10x/0.5 M27 75 mm_4934000013 objective lens at 15x magnification, and ZEN software was used to collect data (Carl Zeiss). Fluorescence was excited with a 488 nm laser, and emission was detected using a 505-545 nm band filter. Post-image processing was performed using Imaris (Oxford Instruments, Abingdon, UK).

### Immunohistochemistry and confocal microscopy

Immunohistochemistry was performed as previously described (Lee et al. 2015) with some modifications. Embryos were fixed in 4% paraformaldehyde (PFA), pH 7.2, at 4°C overnight and dehydrated stepwise in 100% methanol for storage at –20°C. Fixed embryos were rehydrated, incubated in phosphate-buffered saline (PBS) solution containing 0.5% Triton X-100, and blocked in PBS containing 5% sheep serum, 1% bovine serum albumin (BSA), 1% DMSO, and 0.1% Triton X-100 for 1 h at 23°C, and incubated in blocking solution with mouse anti-acetylated α-tubulin monoclonal antibody (Sigma-Aldrich,T-6793; dilution 1:300) for 20 h at 4°C. Embryos were washed in blocking solution without serum and then treated with goat anti-mouse Alexa Fluor-488 secondary antibody (Invitrogen, Waltham, MA, USA; dilution 1:300) at 4°C overnight. Embryos were washed with PBS/0.5% Triton X-100 and incubated in 1x Tris-buffered saline containing SYTO17 red fluorescent nucleic acid stain (Invitrogen S7579; dilution 1:700) for 30 min at room temperature for nuclear staining. Next, embryos were washed in Tris-buffered saline, mounted in glycerol, and imaged using an LSM780 NLO confocal microscope with a 40xwater immersion objective lens (Carl Zeiss) (Kwak et al. 2013). Acetylated tubulin labelled with Alexa Fluor-488 was detected within a range of 491–550 nm following fluorophore excitation by a 488 nm Ar laser, and SYTO 17 red fluorescence was detected at 568–656 nm following excitation by a 561 nm HeNe laser.

### Streptavidin-Cy3 staining

Streptavidin-Cy3 staining was performed as previously described (Sadler et al. 2005) with some modifications. Embryos at 6 dpf were fixed in 4% PFA overnight, washed with PBST (1x PBS, 0.1% Tween), dehydrated through a graded methanol series in PBST, and then stored in 100% methanol at –20°C. On the day of the experiment, embryos were rehydrated through a graded methanol series to PBST, washed thrice with 0.5x saline-sodium citrate (SSC) buffer for 5 min each, bleached with 10% H_2_O_2_/0.5X SSC/0.5% formamide for 12 min, and washed thrice with PBST for 5 min each. Embryos were blocked with PBST/10% BSA for 1 h at 23°C, incubated with CY3-SA (Sigma-Aldrich; dilution 1:500) diluted in the blocking solution in the dark for 2 h at 28°C, washed with PBST, and imaged by confocal microscopy in 80% glycerol after a graded series of glycerol treatment.

### Sectioning and histological analysis

5-week-old fish (*n *= 21) were anaesthetized with 0.02% tricaine, and an incision was made along the ventral midline from the anal pore to just below the gills to allow better penetration of the fixative inside the body cavity. Fish were then fixed in 4% PFA at 4°C for 24 h, dehydrated gradually in ethanol, and embedded in paraffin. Sections (5–10 µm) were cut using a RM2245 microtome (Leica Biosystems, Nussloch, Germany). Tissue sections were stained with haematoxylin-eosin (Sigma-Aldrich), and the sections on the glass slides were scanned using an Axio Scan Z1 slide scanner (Carl Zeiss) or imaged using an Axio Imager A2 microscope with an AxioCam HRc camera (Carl Zeiss).

### Statistical analyses

Statistical analysis was performed using SPSS Statistics 24.0 (IBM Corp., Armonk, NY, USA). Difference between groups was analysed using the Student’s t-test. The threshold for statistical significance for all analyses was set at *P*< .05. Asterisks above a single sample denote comparisons between the sample and control. All quantified zebrafish data are presented as the mean ± standard error of the mean (SEM). The experiments were repeated at least three times.

## Results

### Laterality defects during early embryogenesisin zebrafish

**Figure 1.**
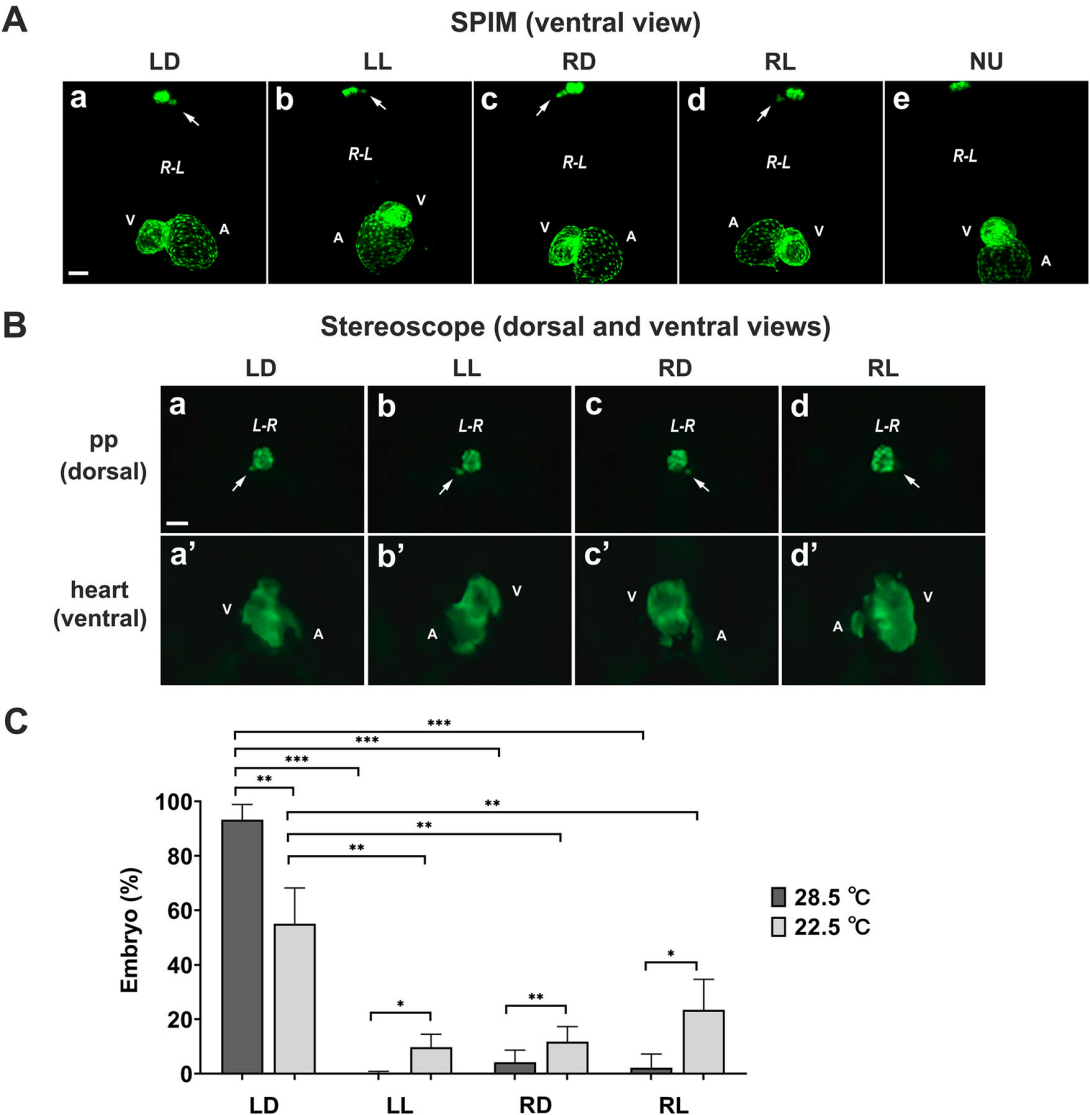
**Laterality defects of the brain and heart in zebrafish embryos. (A)** Ventra lviews of the parapineal (arrow) and the myocardium in 48 hpf *Tg(foxd3:EGFP/myl7:EGFP)* embryos (a–e) visualized using 3D SPIM, which revealed their laterality defect at the cellular level. 10× lens; 1.5× zoom; scale bar, 50 µm. **(B)** Dorsal views of left-right asymmetry of the pineal complex (a–d) and ventral views of the heart (a’–d’) were imaged at 4 days post-fertilization using a fluorescent stereomicroscope. Scale bar, 50 µm. **(C)** Embryos treated with and without cold shock at 22.5°C (*n *= 226) and 28.5°C (*n *= 795), respectively, were examined for their laterality defects of the parapineal (arrow) and cardiac looping at 72hpf. LD, left parapineal(L)and dextral heart looping (D); LL, left parapineal(L)and leftward heart looping (L); RD, right parapineal (R) and dextral heart looping (D); RL, right parapineal (R) and leftward heart looping (L); NU, no parapineal (N) and unloop heart (U); pp, parapineal; *R-L*, right-left sides; *L-R*, left-right sides; A, atrium; V, ventricle. *P< .05, **P< .01, and ***P< .001 by Student’s t-test.

To investigate the aetiology of heterotaxy that occurs during zebrafish development, we initially observed the laterality defects of the pineal complex in the brain and heart looping in 48 hpf zebrafish embryos. The developing pineal complex and myocardium were visualised at the cellular level in the embryos of *Tg(foxd3:EGFP)* and *Tg(myl7:EGFP)* transgenic lines, respectively, using 3D SPIM. 3D imaging revealed the randomization of left-right asymmetry for both organs simultaneously (Figure 1(A)a-d). The combinational orientation related to the parapineal organ and heart was also evaluated at lower resolution using fluorescence microscopy (Figure 1(B)), suggesting a routine screening method for abnormal left-right asymmetry of both pineal complex and heart in 48 hpf embryos. The frequency of the laterality defect phenotype in the wild-type zebrafish was measured as 0.24% left-leftward (LL), 4.20% right-dextral (RD), and 2.24% right-leftward (RL) orientation of the parapineal-heart looping at 50 hpf, while 93.32% embryos showed normal left-dextral (LD) parapineal-heart looping (Figure 1(C)). Then, the impact of temporary environmental change at low temperature on the randomization of laterality was investigated. The embryos were treated with cold shock at 22.5°C from the 16-cell stage (1.5 hpf) to the 16-somite stage (17 hpf) and transferred back to a 28.5°C incubator until examination. The laterality defect of the embryos increased to a frequency of 9.72% left-leftward (LL), 11.74% right-dextral (RD), and 23.45% right-leftward (RL) orientation of parapineal-heart at 72 hpf, while the frequency of embryos with normal left-dextral (LD) orientation was 55.09% (Figure 1(C)). The unloop heart was rarely observed upon cold shock and there was no parapineal (NU) (Figure 1(A)e). These results indicate that the frequency of the laterality change between the pineal complex and heart was in general similar. These results suggest that low temperature may disrupt the asymmetrical patterning of visceral organs during early embryogenesis.

### Hypoplastic endocardial chambers in the embryos with cardiac looping defects

**Figure 2.**
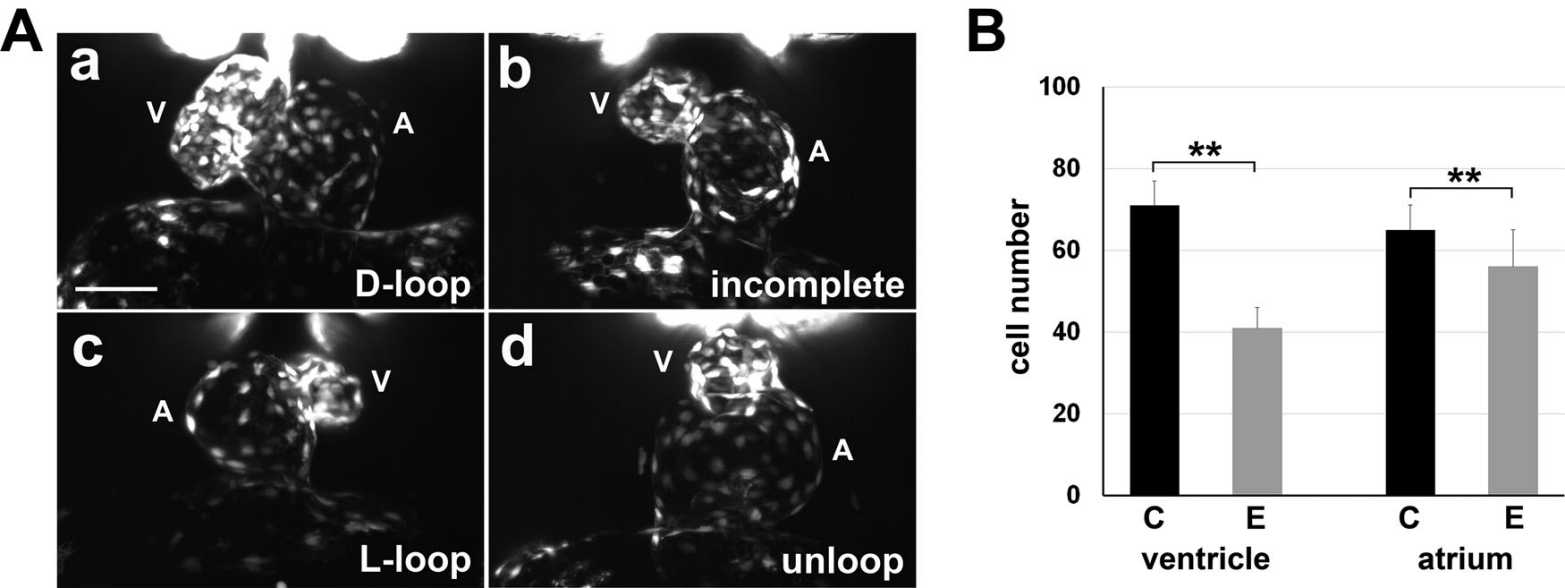
**Cardiac looping defects and hypoplastic ventricle.** (A) 3D SPIM imaging of the developing endocardium of *Tg(fli1:EGFP)* zebrafish embryos revealed cardiac defects in 50 hpf embryos from multiple in-crosses and cold shock: D-loop (a), incomplete loop (b), L-loop (c), and unloop (d) hearts. A, atrium; V, ventricle. Scale bar, 50 µm. **(B)** Total cell number in the endocardium of *Tg(fli1:EGFP)* zebrafish embryos at 50 hpf was counted using the 3D images (*n *= 6 of embryos with D-loop, *n *= 17 of embryos with laterality defects). Endocardial ventricles show more severe hypoplasia in the experimental embryos (E) compared with that in controls (C). Values are presented as mean ± SEM of total cell numbers. **P< .001 by two-sample Student’s *t-*test.

We observed an increase in cardiac looping defect incidence in the embryos obtained from the fish that underwent four or five consecutive generations of inbreeding and were treated with cold shock. Thus, cardiac looping defect was examined in 48 hpf embryos by RNA *in situ* hybridization, using a probe specific for the myocardium marker *myl7*. Overall, 17% and 9% of the embryos showed L-loop and unloop defects, while 44% had a normal D-loop and 30% had an incomplete loop (*n *= 98). The looping defects were visualized in greater detail in 50 hpf larvae of *Tg(fli1:EGFP)* zebrafish, in which endocardial cells express EGFP (Figure 2(A)). 3D SPIM imaging of the heart and cell counting demonstrated that the endocardial chambers of the embryos with cardiac looping defects were hypoplastic and that the ventricles were affected more severely than the atriums at this stage (Figure 2(B))

### Heterotaxy in early zebrafish organ development

**Figure 3.**
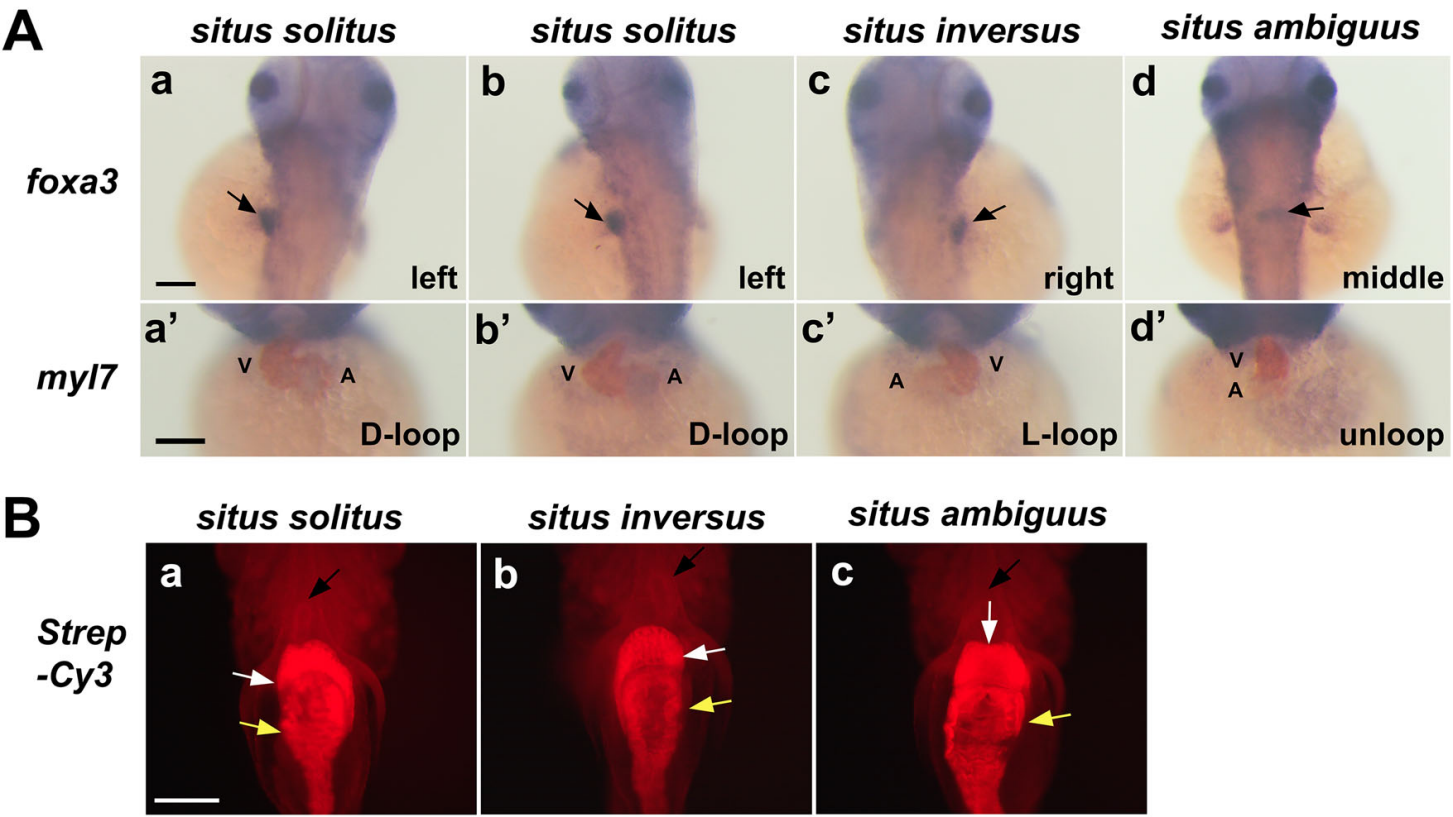
**Situs solitus, situs inversus, and situs ambiguus in zebrafish visceral organ development (A)** Whole mount double *in situ* hybridization reveals laterality of the developing liver (arrow) using a foxa3 RNA probe (purple) in the dorsal view of control (a) and experimental embryos (b–d) and the cardiac looping using the myocardial marker *myl7* (brown) in the ventral view of control (a’) and experimental embryos (b’–d’). A, atrium; V, ventricle. Scale bars, 150 µm. **(B)** Ventral views of 6 dpf larvae stained with Streptavidin-Cy3 (Strep-Cy3) to visualize situs solitus (a), situs inversus (b), and situs ambiguus (c) of the heart (black arrow), liver (white arrow) and gut (yellow arrow). Scale bar, 150 µm.

The heterotaxy phenotype was confirmed with regard to the orientation of the liver and heart in 48 hpf embryos from the multiple inbreeding and cold shock by double *in situ* hybridization using *foxa3* and *myl7* anti-sense RNA probes, respectively (Figure 3(A)). Situs ambiguus embryos with a centred liver and cardiac unloop were observed at frequencies up to 10%, depending on the experimental conditions (Figure 3(A), d and d’). Streptavidin-Cy3 staining of 6 dpf larvae (n=41) revealed situs solitus (63%), situs inversus (27%), or situs ambiguus (10%), indicating the position of the liver, intestine, and heart in each larva (Figure 3(B)). These data demonstrate that the experimental manipulation may disrupt the asymmetrical patterning of visceral organs during early zebrafish embryogenesis, thereby resulting in heterotaxy.

### Altered nodal signalling and Kupffer’s vesicle formation

**Figure 4.**
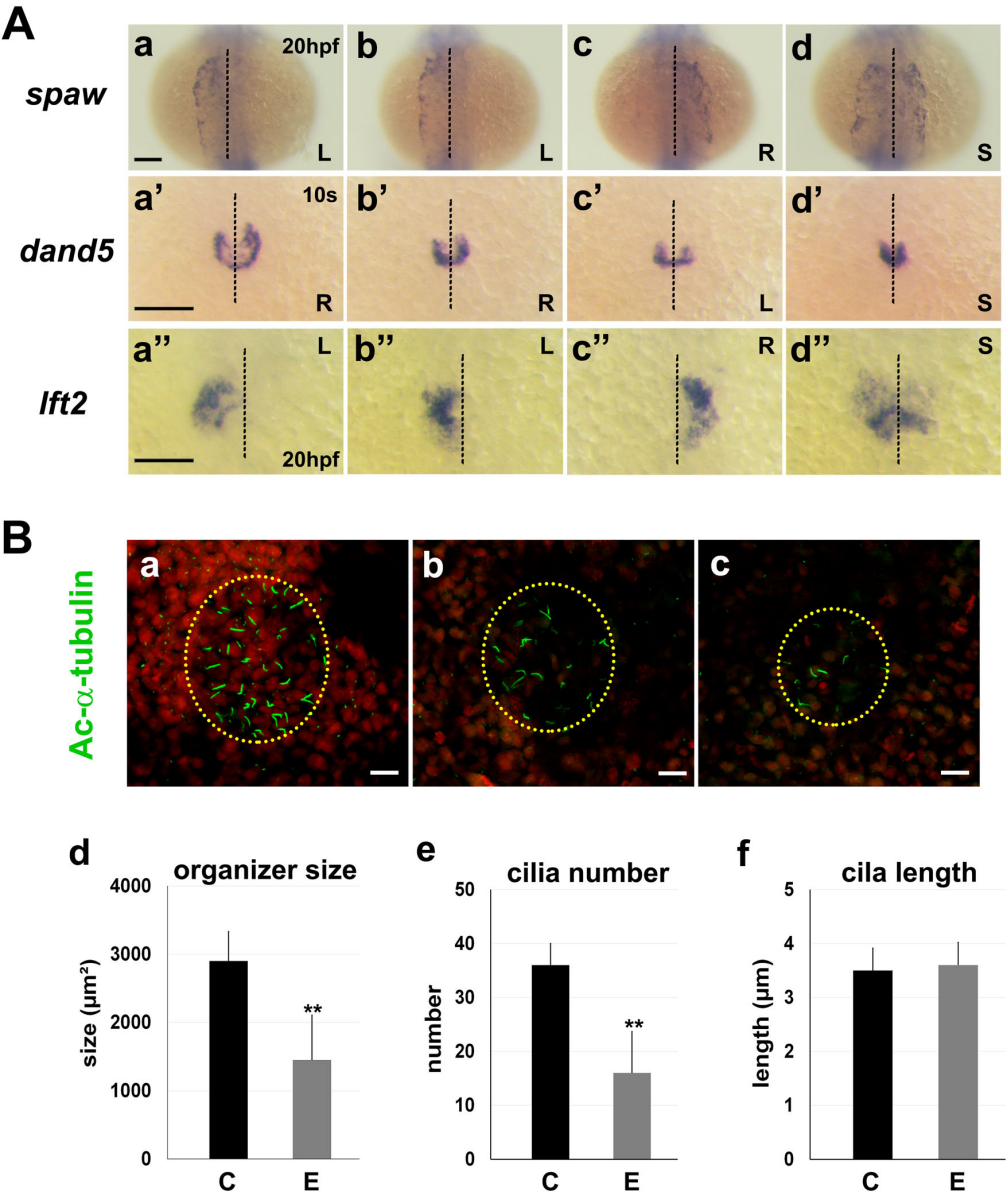
**Disruption of the nodal signalling and left-right organizer development in zebrafish embryos in the experimental conditions of over-crosses and temporary cold shock during early development.** **(A)** *In situ* hybridization reveals defect in nodal signalling. Asymmetrical expressions of *dand5, spaw* and *lft2* are often randomized in the experimental embryos (b-d, b’-d’, and b”-d”) compared to controls (a, a’, and a”) respectively. B, bilateral; L, left-sided; R, right-sided; S, symmetrical. Scale bars, 150 µm. **(B)** The experimental condition causes reduced size of the Kuffer’s vesicle and lower number of cilia. (a-c) Confocal imaging showing primary cilia (green) labelled with an anti-acetylated α-tubulin (Ac-a-tubulin) antibody, Kuffer’s vesicle (yellow dotted circle), and the nuclei of neighbouring cells (red, SYTO17 staining). Scale bar, 20 µm. (d-f) Organizer size, cilia number and the cilia length were compared between the control (C) and experimental embryos (E). Statistical difference between groups was evaluated by the Student’s t-test.­­ **P< .0005.

Whether altered nodal signalling could contribute for the observed heterotaxic phenotypes in the abovementioned experimental conditions was examined next. *In situ* hybridization experiments showed that the expression of nodal signalling-associated transcripts around Kupffer’s vesicle, the main left-right organizer, was disrupted in the experimental embryos. The nodal homolog *spaw*, normally expressed around the Kupffer’s vesicle and in the left lateral plate mesoderm leading to left-specific morphogenesis, was observed to be expressed in the right lateral plate mesoderm or bilaterally in the 46% of experimental embryos at 20 hpf (*n *= 46) (Figure 4(A), a–d). Similarly, expression of *dand5*, a nodal inhibitor expressed more on the right side around Kupffer’s vesicle (Figure 4(A), a’-d’) and *lft2*, a nodal downstream gene expressed in the left lateral plate mesoderm and heart field (Figure 4(A), a’’-d’’), were often both randomized in the experimental embryos at 10 somite stage (ss) and 20 hpf, respectively, implicating that nodal signalling may underlie the left-right patterning defect.

Since the region in which *dand5* was expressed around Kupffer’s vesicle was often reduced in embryos from fish that underwent the experimental conditions of over-crosses and temporary cold shock in early development (Figure 4(A), a’-d’), it was reasonable to hypothesize that abnormal development of Kupffer’s vesicle and subsequently altered nodal signalling may contribute for laterality defects. To test this hypothesis, the size of the Kupffer’s vesicles and the number of associated cilia in control (*n *= 8) and experimental (*n *= 28) embryos were determined by immunohistochemistry of 6-somite stage embryos with anti-acetylated α-tubulin labelling of primary cilia and subsequent confocal microscopy imaging (Figure 4(B), a-c). The Kupffer’s vesicles of the experimental embryos were smaller and contained fewer cilia than those of control embryos, while cilia length was similar between the two groups (Figure 4(B), d-f).

### Characterization of the heterotaxy in juvenile fish

**Figure 5.**
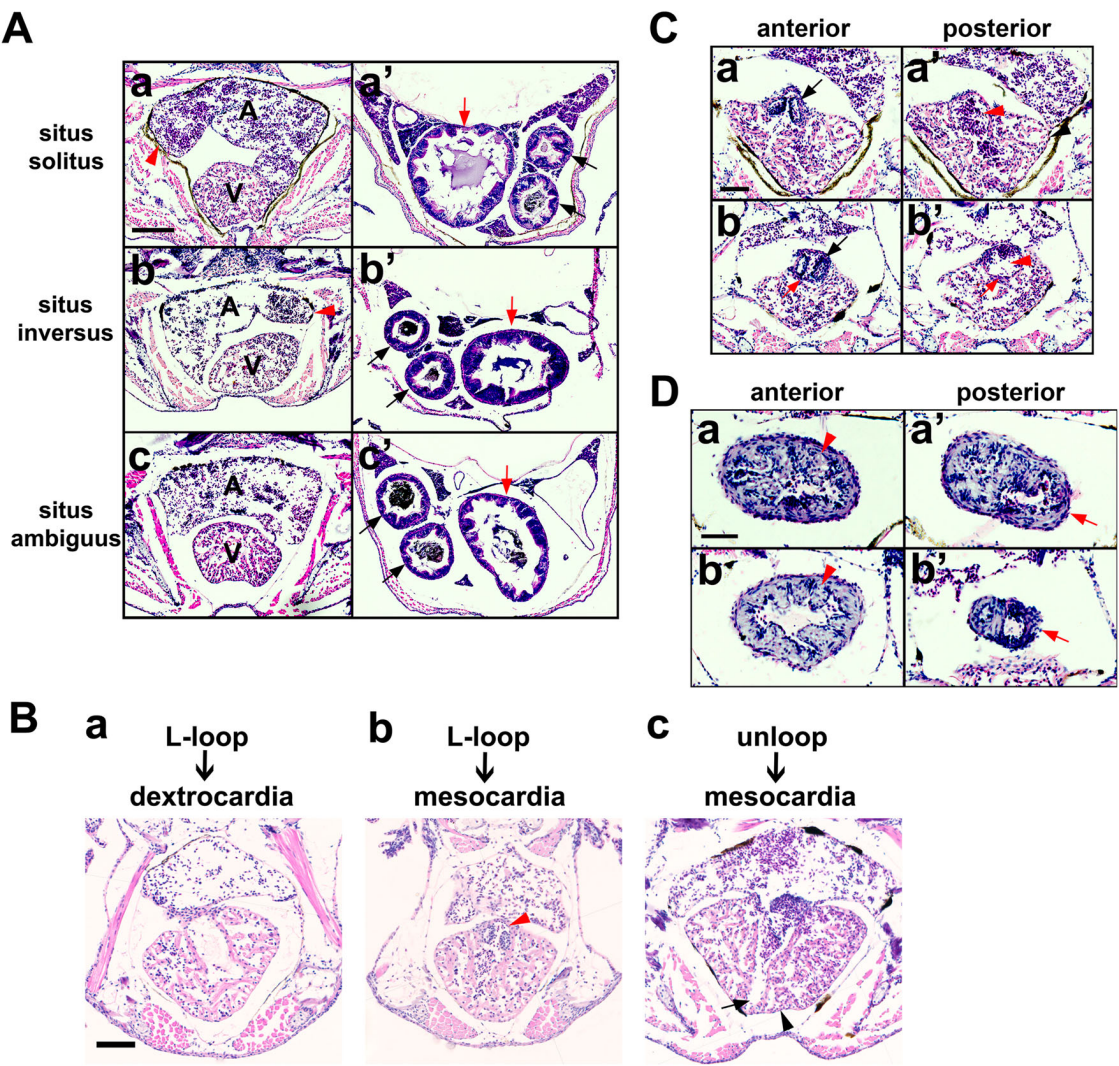
**Histopathological assessment of heterotaxy in juvenile zebrafish. (A)** Haematoxylin and eosin–stained transverse paraffin sections of 5-week-old zebrafish showing situs solitus, situs inversus, and situs ambiguus of the heart (a–c) and other visceral organs (a’–c’) in each fish. Atrium tip, red arrowhead; intestinal bulb, red arrow; two anterior and posterior intestines, black arrows. Malrotated guts (arrows) are shown in the fish. Scale bars, 100 µm. Laterality of visceral organs in the selected fish are summarized in Table 1. **(B)** L-looped heart in 48 hpf embryo developed to dextrocardia (a) or mesocardia (b) at 5 weeks of age, while unlooped heart developed to mesocardia (c). Additionally, some experimental fish had transposition of the bulbo-ventricular valve (b, red arrowhead), non-apex ventricle (c, black arrowhead), and abnormal atrioventricular canal (c, black arrow). Scale bar, 100 µm. **(C)** Abnormal structure and laterality defect of the outflow tract (bulbus arteriosus–ventricle) valve (black arrow), blood flow defect in the outflow tract (red arrowhead), and ectopic membrane (red arrow) are shown in Fish #12. Scale bar, 50 µm. **(D)** Coarctation of the outflow tract (arrow) with defective smooth muscle layer (arrowhead) was observed in Fish #12. Scale bar, 50 µm.

To monitor whether the early embryonic laterality defects were maintained in juvenile fish, the embryos were sorted according to the direction of their cardiac looping at 2 dpf embryonic stage and raised until age 5-weeks. Histological analysis of the transverse sections revealed laterality defects in the visceral organs of the fish: situs solitus, situs inversus, and situs ambiguus (Figure 5; Table 1).

**Table 1.** Histopathological assessment of situs solitus, situs inversus, and situs ambiguus in juvenile fish.

Fish #	gender	48hpf cardiac looping	Juvenile organ situs	heart	intestinal bulb	intestine	pancreas	gall bladder	spleen
1	♂	D-loop	situs solitus	L	R	L	R	R	L
2	♀	D-loop		L	R	L	R	R	ND
3	♂	D-loop		L	B	B	R	R	L
4	♂	D-loop		L	R	L	R	R	L
5	♂	D-loop		L	R	L	R	R	L
6	♂	D-loop		L	B	B	R	R	L
7	♀	D-loop		L	R	L	R	R	L
8	♂	D-loop		L	R	L	R	R	L
9	♂	D-loop		L	B/R	L	R	R	L
10	♂	D-loop		L	R	L	R	R	ND
11	♂	L-loop	situs inversus	R	L	R	L	L	R
12	♀	L-loop		R	L	R	L	L	R
13	♂	L-loop		R	L	R	L	L	R
14	♂	L-loop	situs ambiguus	B	B/L	R	L	L	R
15	♂	L-loop		B	B	B	L	L	R
16	♂	L-loop		R	R	L	L	L	R
17	♀	unloop		B	L	R	L	L	R
18	♀	unloop		R	R	L	L	L	R
19	♀	D-loop	situs solitus	L	R	L	R	R	L
20	♂	D-loop		L	R	L	R	R	L
21	♂	I-loop		L	R	L	R	R	L

Note: The laterality of visceral organs was assessed by haematoxylin and eosin staining of 5-week-old juvenile fish selected according to their cardiac looping morphology of the 48 hpf control or experimental embryo. Ten experimental fish (#1–#10) were grown from embryos with D-looped heart; six (#11–#16) from embryos with L-looped heart; two (#17, #18) from embryos with unlooped heart. Three fish (#19–#21) were grown from control embryos with D-loop or incomplete loop. B, bilateral; D-loop, dextral looping; F, female; I-loop, incomplete looping;L, left-sided; L-loop, leftward looping; M, male; ND, not determined; R, right-sided.

Among the 21 fish examined (Table 1), six embryos originally with L-looped heart at 48 hpf, developed the organ laterality of situs inversus (Fish #11–13; Figure 5(A)) or situs ambiguus (Fish #14–16; Figure 5(A)) at 5 weeks, while 10 embryos with D-looped heart developed situs solitus (Fish #1–10; Figure 5(A)), among which two showed incomplete intestinal rotation (Fish #3 and #6). One of the two embryos with unlooped hearts developed into 5-week-old fish with mesocardia and inversion of visceral organs (Fish #17; Figure 5(B)) and the other with randomization of laterality of the examined organs (Fish #18). Three control embryos with D-looped heart or incomplete loopingat 48 hpf developed situs solitus at 5 weeks (Fish #19–21). These results suggest that differential compensation may occur during organ development after initial cardiac looping.

Further analysis revealed the following phenotypes: non-apex forming ventricle (Figure 5(B)), transposition of the outflow tract (Figure 5(B)), structural malformation of the bulbo-ventricular valve surrounded by an ectopic membrane resulting in interruption of blood outflow from the ventricle (Figure 5(C)), coarctation of the outflow tract (Figure 5(D)), and reduced smooth muscle layer (Figure 5(D)). These results suggest that a small number of embryos may experience abnormal neural crest development, in addition to alteration of left-right asymmetry.

## Discussion

Heterotaxy syndrome is a congenital disorder resulting from the disruption of the left-right body patterning. Most patients with heterotaxy have serious congenital heart diseases, that are associated with poor survival rates and outcomes. However, the underlying aetiology and mechanisms in the majority of heterotaxy cases remain unknown. This study explored the laterality defects of the visceral organs in zebrafish during development, which were induced by experimental conditions, resulting in anatomical aberrations in the larvae and juvenile fish, thereby recapitulating the symptoms of heterotaxy.

3D SPIM was used to visualise at the cellular level the randomization of left-right asymmetry of both the parapineal and heart organs in an organism, suggesting that it is a valuable tool to simultaneously evaluate the laterality defects of different combinations of visceral organs in a single organism (Figure 1(A)). The combinational orientation of the parapineal organ and the heart was also evaluated by separate imaging of the two organs at lower resolution using a fluorescence microscopy (Figure 1(B)). Both the bright field and fluorescence microscopy are good enough to select embryos with cardiac looping defects at 48 hpf and onward for routine screening of abnormal left-right asymmetry in zebrafish.

Abnormal development of left-right asymmetry in the brain contributes to neuropathological disorders; thus, it is essential to understand the basis underlying the perturbation of the left-right asymmetry and define early morphological markers. The combined results from the present experiments showed that the frequency of laterality changes of the parapineal and cardiac looping was in general similar, indicating that the laterality development of the pineal complex and heart may share the common signalling events temporarily or spatially in the early development. The mechanism underlying the establishment of the left-right asymmetry during embryogenesis is conserved among vertebrates. The asymmetric development of the visceral organs is controlled largely by *nodal* (Long et al. 2003; Schier 2009), a transforming growth factor-β protein that is asymmetrically directed by motile cilia in the left-right organizer to the lateral plate mesoderm during early embryonic development (Essner et al. 2005; Matsui and Bessho 2012). Here in, disruption of nodal signalling was found to underly randomization of the left-right asymmetry of the parapineal and cardiac looping defects in the inducible experimental condition (Figure 4(A)).

The histopathological assessment of situs solitus, situs inversus, and situs ambiguus using a zebrafish model may advance the research on heterotaxy. This study demonstrates that early visualization of cardiac looping defects may represent a useful marker to select zebrafish with laterality defects, which can in turn be used to investigate the histopathology of left-right asymmetry of visceral organs in long-term studies or to benefit the research by reducing the animals with a background of laterality defects from the model animals.
